# Increasing Time in Therapeutic Range of Tacrolimus in the First Year Predicts Better Outcomes in Living-Donor Kidney Transplantation

**DOI:** 10.3389/fimmu.2019.02912

**Published:** 2019-12-20

**Authors:** Turun Song, Saifu Yin, Yamei Jiang, Zhongli Huang, Jinpeng Liu, Zhiling Wang, Linde Li, Jun Zeng, Yu Fan, Xianding Wang, Xingxing Li, Tao Lin

**Affiliations:** Urology Department, Urology Research Institute, Organ Transplantation Centre, West China Hospital, Sichuan University, Chengdu, China

**Keywords:** tacrolimus, time in therapeutic range, kidney transplantation, development and validation, acute rejection, infection, graft loss, patient death

## Abstract

**Background:** The aim of the present study was to investigate the impact of time in therapeutic range TTR on long-term outcomes of living kidney transplants.

**Methods:** We included 1,241 living kidney transplants and randomized them into development and validation cohorts with a ratio of 2:1. The tacrolimus TTR percentage was calculated by linear interpolation with a target range (5–10 ng/ml months 0–3, 4–8 ng/ml months 4–12). The optimal TTR cutoff was estimated by the receiver operating characteristic curve analysis on the basis of acute rejection (AR) within 12 months in the development cohort. Outcomes were analyzed between patients with high TTR and low TTR in the development and validation cohorts, respectively. The TTR was also compared with other tacrolimus measures.

**Results:** The optimal TTR cutoff value was 78%. In the development cohort, patients with TTR > 78% had significantly higher rejection- and infection-free survival. TTR < 78% was an independent risk factor for AR (OR: 2.97, 95%CI: 1.82–4.84) and infection (OR: 1.55, 95%CI: 1.08–2.22). Patient and graft survival were significantly higher in those with TTR>78%, and TTR<78% was associated with graft loss (OR: 3.2, 95%CI: 1.38–7.42) and patient death (OR: 6.54, 95%CI: 1.34–31.77). These findings were confirmed in the validation cohort. Furthermore, we divided all included patients into a high and low TTR group. TTR was more strongly associated with patient and graft survival than mean level, standard deviation, and intrapatient variability (IPV).

**Conclusions:** Increasing the TTR of tacrolimus in the first year was associated with improved long-term outcomes in living kidney transplants, and TTR may be a novel valuable strategy to monitor tacrolimus exposure.

## Introduction

Tacrolimus-based regimens are the most commonly used immunosuppressive therapies, preventing T-cell and antibody-mediated rejection after kidney transplantation (1); however, a narrow therapeutic index limited their clinical application. Overexposure can result in toxicity and severe infection, and underexposure can lead to graft rejection (2, 3). Therapeutic drug monitoring to maintain the intensity and stability and a constant tacrolimus trough level allows for the achievement of optimal immunosuppression. Mean level, intrapatient variability (IPV), and variability of the standard deviation of the tacrolimus trough level are associated with acute rejection (AR) and graft loss (4–6). However, these indexes did not consider whether the tacrolimus trough level achieved a target therapeutic window and exposure time. As failure to maintain the tacrolimus trough level in target ranges is a risk factor for inferior short- and long-term outcomes (7, 8), it seems more practical and clinically relevant to develop a new indicator to combine the tacrolimus trough level with variation and the corresponding maintaining time.

Percent time in therapeutic range (TTR), defined as the percentage of time within the therapeutic range over time, takes stability, intensity, and constancy into consideration simultaneously. TTR is a validated method for assessing effective warfarin therapy and has been used as a tool to risk stratifying (9, 10). Limited studies have investigated the use of TTR in transplantation, but a low tacrolimus TTR was associated with significantly increased acute cellular risk in lung transplants (11) and *de novo* donor-specific antibodies (dnDSAs) in kidney transplants (12). These facts indicated that the tacrolimus TTR might have potential as a prognostic indicator in organ transplantation, but evidence of its impact on the long-term outcomes of living kidney transplants is lacking. The present study investigated whether patients with a high tacrolimus TTR had better clinical results than those with a low tacrolimus TTR.

## Methods

### Patient Population

The clinical data of patients who received a living-related kidney transplant at West China Hospital between August 2007 and April 2017 were retrospectively analyzed. The Ethics Committee of West China Hospital approved the study. Patients who were <18 years of age, with an initial calcineurin inhibitor (CNI) other than tacrolimus, tacrolimus switch or withdrawal in first 12 months, receiving an ABO-incompatible kidney transplant, with organ transplant history, a follow-up of <1 year, or with three or more consecutive missing measures of tacrolimus trough level, according to our monitoring protocol, were excluded.

### Data Collection

We retrieved information from medical records, including patient age, sex, body mass index at the time of transplantation, duration of pretransplantation dialysis, organ transplant history, panel reactive antibody (PRA), human leukocyte antigen (HLA) mismatch, induction therapy, delayed graft function (DGF), and cold ischemic time. DGF was defined as the need for dialysis in the first week after transplantation. AR, infection, graft loss, and patient death were the clinical outcomes of interest. AR was diagnosed clinically based on a 50% or more significant increase in serum creatinine levels within 3 days that was not explained by some other cause and that was confirmed by biopsy when necessary. AR was treated primarily with bolus doses of methylprednisolone and with antithymocyte globulin if refractory. Infection was defined as any infectious symptoms needing medication intervention, including wound, pulmonary, urinary tract, and skin infections. Re-establishment of long-term dialysis therapy or estimated glomerular filtration rate (eGFR) of <15 ml/min was considered as graft loss. Allograft survival was censored at the earliest of the following events: loss to follow-up or patient death. The definition of graft failure did not include patient death with a functioning graft. Renal function was assessed by eGFR, calculated using the Modification of Diet in Renal Disease (MDRD) equation for Chinese and adjusted for body surface area (13).

### Immunosuppression Regimen

The immunosuppression therapy used at our hospital has been previously described (14). Briefly, rabbit antihuman thymocyte immunoglobulin (ATG) (1 mg/kg administered for 3–7 days) or monoclonal anti-CD25 monoclonal antibody (IL-2R antibody) (20 mg on days 0 and 4 post-transplant) were used as induction therapy. Maintenance immunosuppressive therapy consisted of tacrolimus, mycophenolate mofetil/enteric-coated mycophenolate sodium (MMF/EC-MPS), and corticosteroids. Tacrolimus was initiated at 1.5 mg bid on day two post-transplantation and maintained at 5–12 ng/mL. The tacrolimus trough level was measured by the enzyme multiplied immunoassay technique (EMIT, Dade-Behring, NY, USA) in blood samples collected weekly during months 0–3, every 2 weeks during months 4–6, and monthly thereafter in the first year. Tacrolimus trough levels were obtained before breakfast and dose administration in the morning. Any tacrolimus levels that were <2 or > 15 ng/mL were individually reviewed and excluded if they were not valid. MMF was started on the night before the operation at 1,000 mg and maintained at 1,000 mg bid. The area under the curve (AUC) for mycophenolate mofetil was 30–70 mg/h·L^−1^. EC-MPS 720 mg was administrated the night before the operation and at 720 mg bid after that. The EC-MPS AUC was not measured. Methylprednisolone 500 mg was administered intravenously during the surgery, and 300 mg was given daily for the next 3 days. It was then replaced by 60 mg of prednisone, which was tapered by 10 to 5–10 mg/day for maintenance.

### Time in Therapeutic Range

We randomized all patients into development and validation cohorts with a ratio of 2:1. The process was finished by SPSS 24.0 software. First, SPSS gave each individual a random number. Then, these number were ranked in order from large to small. Last, the first 2/3 was used as the developed cohort and the latest 1/3 was used as the validation cohort. The tacrolimus TTR percentage was calculated by linear interpolation as described by Rosendaal in the development cohort (15). The linear relationship between each tacrolimus trough level and the TTR was calculated by summing the time during which the value fell within the target tacrolimus range of 5–10 ng/ml during months 0–3 and 4–8 ng/ml during months 4–12. The tacrolimus TTR was compared with the tacrolimus standard deviation (SD), mean, and the IPV. The IPV was calculated by dividing SD by the mean level (16).

### Statistical Analysis

Descriptive statistics were used to describe the baseline characteristics of the patients in the development and validate cohorts. Categorical variables were compared using the χ^2^-test or Fisher's exact test; continuous variables were compared using a Student's *t*-test. A receiver operating characteristic (ROC) curve was built from the calculated TTR value in development cohort to determine the optimal TTR cutoff value that can discriminate patients with or without AR in the first year best. The area under the ROC curve with sensitivity and specificity was computed, and the TTR with the greatest AUC is the optimal cut-off value (17).

Time to AR, infection, graft loss, and recipient death was analyzed by the Kaplan–Meier method, and between-group differences were assessed for significance by the log-rank test. Cox Proportional regression was used to identify predictors of AR, infection, graft loss, and patient death. Variables with *p* < 0.1 in the univariate analysis were included in the multivariate analysis. The statistical analysis was performed using SPSS 24.0 (IBM Corp., Armonk, NY, USA). *P* < 0.05 was considered significant.

## Results

From August 2007 to April 2017, 2,048 patients received a living related kidney transplant in West China Hospital, Sichuan University. A total of 807 patients were excluded: follow-up time was <1 year (*N* = 227); CNI was not tacrolimus (*N* = 275); lost in the follow-up (*N* = 82); organ transplantation history (*N* = 15); ABO-incompatible kidney transplantation (*N* = 38); and consecutive missing of tacrolimus trough level 3 times or more (*N* = 170). A total of 1,241 patients were included with 827 in the development cohort and 414 in the validation cohort. The process of enrollment of patients is shown in the flow chart (Supplementary Figure 1). The clinical characteristics of the development cohort and validation cohort are shown in Table 1. The median follow-up for the entire cohort was 42 months [interquartile range (IQR): 26–59 months]; 42 months (IQR: 26–59 months) for the development cohort, and 42 months (IQR: 25–59 months) for the validation cohort.

**Table 1:** Clinical characteristics of the development cohort and validation cohort.

**Characteristics^a^**	**Development (*N* = 827)**	**Validation (*N* = 414)**	***P*-value**
Donor age (years)	47.3 (±9.8)	47.4 (±9.7)	0.88
Donor sex (Male)	33.00%	31.50%	0.72
Donor eGFR	109.4 ± 13.7	115.6 ± 17.5	0.63
Recipient age (years)	33.1 (±8.3)	32.7 (±8.7)	0.43
Recipient sex (Male)	71.70%	71.90%	0.95
Recipient BMI, Kg/m^2^	21.5 (±3.5)	21.8 (±18)	0.72
Cold ischemic time (h)	2.5 (±0.9)	2.5 (±0.9)	0.88
DGF	1.10%	1.50%	0.48
Induction therapy			0.46
No	33.90%	31.60%	
ATG	11.95%	10.60%	
IL-2 R antibody	54.20%	57.90%	
HLA mismatches			0.11
≤3 mismatch	11.50%	15.00%	
>3 mismatch	88.50%	85.00%	
Pre-transplant PRA >20%	2.90%	2.90%	0.95
Duration of dialysis, months	13.4 (±14.8)	13.4 (±15.8)	0.94
Transplant year			0.56
2007–2012	266	140	
2013–2017	561	274	

*ATG, antithymocyte globulin; BMI, body mass index; eGFR, estimated glomerular filtration rate; DGF, delayed graft function; HLA, human leukocyte antigen; PRA, panel reactive antibody*.

a *Continuous data are presented as mean ± standard deviation (SD), and categorical data as percentage of the total, unless otherwise noted*.

**Figure 1:**
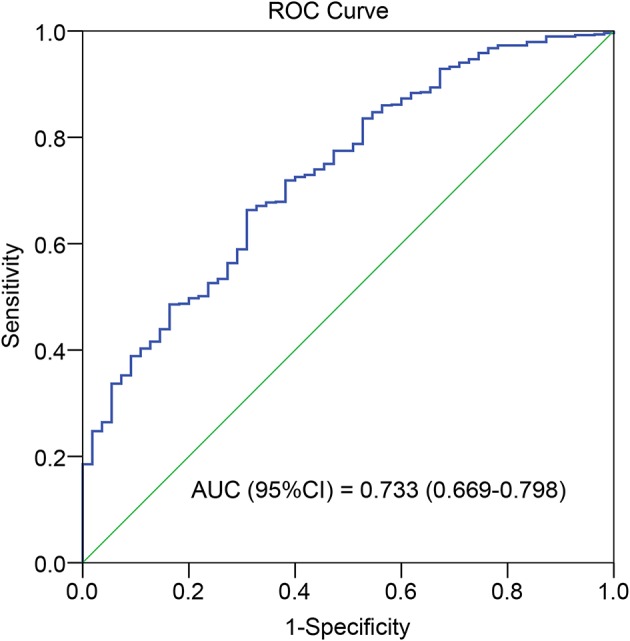
Receiver operating characteristic curve of the TTR.

**Figure 2:**
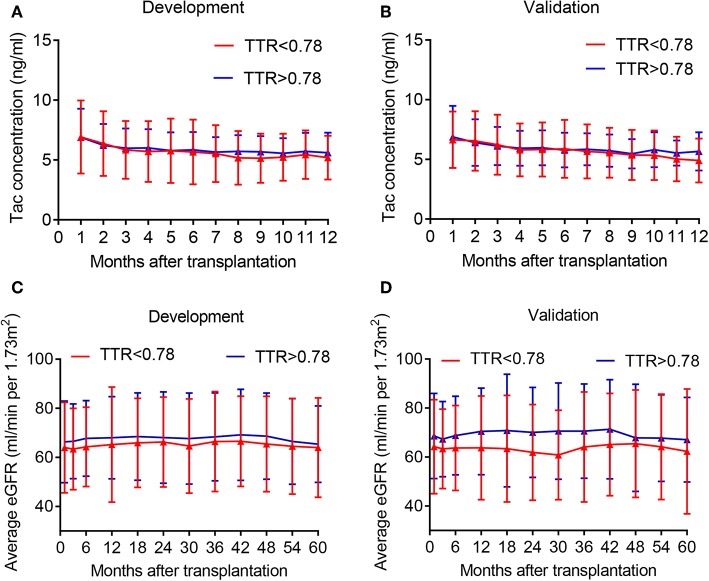
Average tacrolimus concentration and eGFR distribution in the development and validation cohort at different time point. **(A)** Tac concentration in the development cohort; **(B)** Tac concentration in the validation cohort; **(C)** eGFR in the development cohort; **(D)** eGFR in the validation cohort. Tac, tacrolimus.

The optimal cutoff value for TAC TTR was 78% (AUC = 0.733; sensitivity = 66.3%; and specificity = 69.1%) (Figure 1). We thus divided patients in the development and validation cohort into a high TTR group and low TTR group. Baseline characteristics between the high and low TTR groups in both cohorts are summarized in Table 2. All variables were comparable between high and low TTR groups in the development cohort; however, in the validation cohort, patients in the low TTR group received kidneys from older donors (*P* = 0.02) and had more HLA mismatches (*P* = 0.02). There was no difference in the tacrolimus trough levels between high and low TTR groups within the first year in the development and validation cohort. No difference was detected in renal function between the two groups during the follow-ups in the development cohort, while the high TTR group in validation cohort had a litter higher eGFR in first 3 years (mean difference 4~5 ml/min/1.73 m^2^) (Figure 2).

**Table 2:** Characteristics between the high and low TTR groups in development and validation cohorts.

**Characteristics**	**Development**		***P*-value**	**Validation**		***P*-value**
	**TTR <78% (*N* = 283)**	**TTR>78% (*N* = 544)**		**TTR <78% (*N* = 145)**	**TTR>78% (*N* = 269)**	
Donor age (years)	46.8 (±10.2)	47.7 (±9.5)	0.31	46 (±9)	48.5 (±9.8)	0.09
Donor sex (Male)	30.70%	32.70%	0.61	28.20%	36.90%	0.22
Donor eGFR	111.3 ± 11.6	108.4 ± 14.9	0.46	118.6 ± 20.8	114.0 ± 11.8	0.57
Recipient age (years)	32.9 (±8.7)	32.8 (±8.5)	0.87	34.4 (±8.5)	32.4 (±8.1)	0.02
Recipient sex (Male)	72.00%	71.80%	0.95	71.05%	72.10%	0.81
Recipient BMI, kg/m^2^	21.4 (±3)	21.9 (±18)	0.53	21.5 (±3)	21.4 (±3.7)	0.74
Cold ischemic time (h)	2.5 (±0.9)	2.6 (±0.9)	0.13	2.5 (±0.9)	2.6 (±0.9)	0.52
DGF	1.40%	1.00%	0.7	1.30%	1.60%	0.37
Induction therapy			0.21			0.21
No	35.50%	29.70%		37.90%	31.30%	
ATG	9.20%	11.30%		13.80%	10.80%	
IL-2 R antibody	55.30%	59.00%		48.30%	57.80%	
HLA mismatches			0.1			0.02
≤3 mismatch	86.20%	78.70%		82.60%	90.90%	
>3 mismatch	13.80%	21.30%		17.40%	9.10%	
Pre-transplant PRA >20%	3.00%	2.80%	0.95	2.80%	2.60%	0.93
Duration of dialysis, months	13.2 (±14.9)	13.5 (±15.8)	0.76	14.4 (±15.7)	12.8 (±14.3)	0.29
Transplant year			0.53			0.52
2007–2012	87	179		52	88	
2013–2017	196	365		93	181	

*ATG, antithymocyte globulin; BMI, body mass index; eGFR, estimated glomerular filtration rate; DGF, delayed graft function; HLA, human leukocyte antigen; PRA, panel reactive antibody*.

### Graft Survival

**Figure 3:**
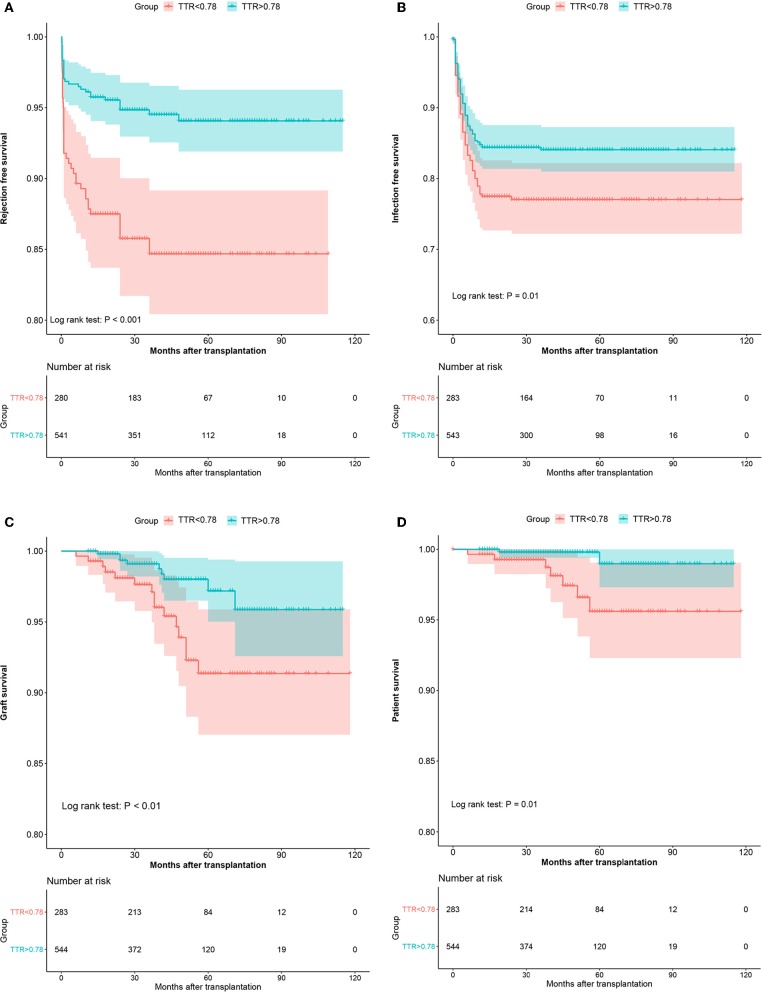
The Kaplan–Meier curves of the rejection-free survival **(A)**, infection-free survival **(B)**, graft survival **(C)**, and patient survival **(D)** in the development cohort.

In the development cohort, more patients experienced graft loss (15/283, 5.3%) in the low TTR group than those in the high TTR (9/544, 1.7%) (*P* < 0.001). The Kaplan–Meier curve indicated that the graft survival in the high TTR group was significantly higher than that in the low TTR group (Figure 3C). The 3- and 5-year graft survival was 97.5 and 96.6% as well as 93.5 and 92.3% for the high and low TTR group, respectively. Lower TTR was an independent graft-loss contributor in the multivariate analysis (OR: 3.2, 95%CI: 1.38–7.42). A younger age also seemed to be protective for graft survival (OR: 0.95, 95%CI: 0.90–1.00) (Table 3).

**Table 3:** Univariate and Multivariate analysis of clinical characteristics for acute rejection, infection, and patient and graft survival in development cohort.

**Characteristic**	**Acute rejection**				**Infection**				**Graft survival**				**Patient survival**			
	**Unadjusted**	***P***	**Adjusted**	***P***	**Unadjusted**	***P***	**Adjusted**	***P***	**Unadjusted**	***P***	**Adjusted**	***P***	**Unadjusted**	***P***	**Adjusted**	***P***
TTR	3.36 (2.21–5.11)	<0.001	2.97 (1.82–4.84)	<0.001	1.55 (1.08–2.22)	0.019	1.55 (1.08–2.22)	0.019	3.33 (1.44–7.7)	0.005	3.2 (1.38–7.42)	0.007	6.87 (1.42–33.31)	0.017	6.54 (1.34–31.77)	0.02
Donor age	1.01 (0.98–1.04)	0.58			1.02 (0.99–1.05)	0.277			1.02 (0.96–1.09)	0.538			1.02 (0.94–1.11)	0.638		
Donor sex	0.67 (0.34–1.32)	0.24			0.59 (0.31–1.13)	0.112			1.27 (0.36–4.43)	0.707			0.44 (0.05–3.78)	0.452		
Recipient age	0.98 (0.96–1.01)	0.17			0.99 (0.97–1.01)	0.196			0.95 (0.9–1)	0.052	0.95 (0.9–1)	0.07	1.04 (0.97–1.12)	0.226		
Recipient sex	1.21 (0.76–1.93)	0.43			1.15 (0.77–1.72)	0.506			1.18 (0.46–3)	0.733			3.15 (0.39–25.35)	0.28		
Recipient BMI	0.99 (0.93–1.05)	0.79			0.99 (0.97–1.02)	0.72			0.95 (0.8–1.13)	0.586			1 (0.98–1.03)	0.812		
Time of dialysis	1 (0.98–1.01)	0.56			1 (0.99–1.01)	0.69			1 (0.98–1.03)	0.687			1.01 (0.99–1.04)	0.291		
DGF	0.21 (0.07–0.7)	0.01	0.21 (0.06–0.77)	0.018	0.43 (0.13–1.44)	0.172				0				0		
HLA mismatch	1.49 (0.7–3.2)	0.3			0.99 (0.59–1.65)	0.974			1.93 (0.45–8.3)	0.379			1.38 (0.17–11.17)	0.761		
PRA	1.17 (0.27–5.06)	0.83			1.15 (0.39–3.4)	0.802			0.71 (0.09–5.47)	0.741				0		
Cold ischemic time	0.73 (0.55–0.95)	0.02	1 (0.98–1.01)	0.91	0.92 (0.75–1.13)	0.427			1 (0.63–1.58)	0.996			0.48 (0.21–1.1)	0.082	0.5 (0.22–1.15)	0.102
Induction therapy		0.15				0.254				0.297				0.838		
No	Reference				Reference				Reference				Reference			
ATG	1.65 (0.99–2.73)				0.81 (0.54–1.22)				0.42 (0.14–1.26)				0.6 (0.12–3.02)			
IL-2R	1.24 (0.55–2.76)				1.33 (0.77–2.32)				0.96 (0.28–3.36)				0.91 (0.11–7.66)			
Transplant year		0.36				0.62				0.48				0.56		
2007–2012	Reference				Reference				Reference				Reference			
2013–2017	0.85 (0.64–1.13)				0.94 (0.76–1.38)				0.89 (0.43–1.84)				0.91 (0.58–1.43)			

*ATG, antithymocyte globulin; BMI, body mass index; eGFR, estimated glomerular filtration rate; DGF, delayed graft function; HLA, human leukocyte antigen; PRA, panel reactive antibody; TTR, time in therapeutic range*.

**Figure 4:**
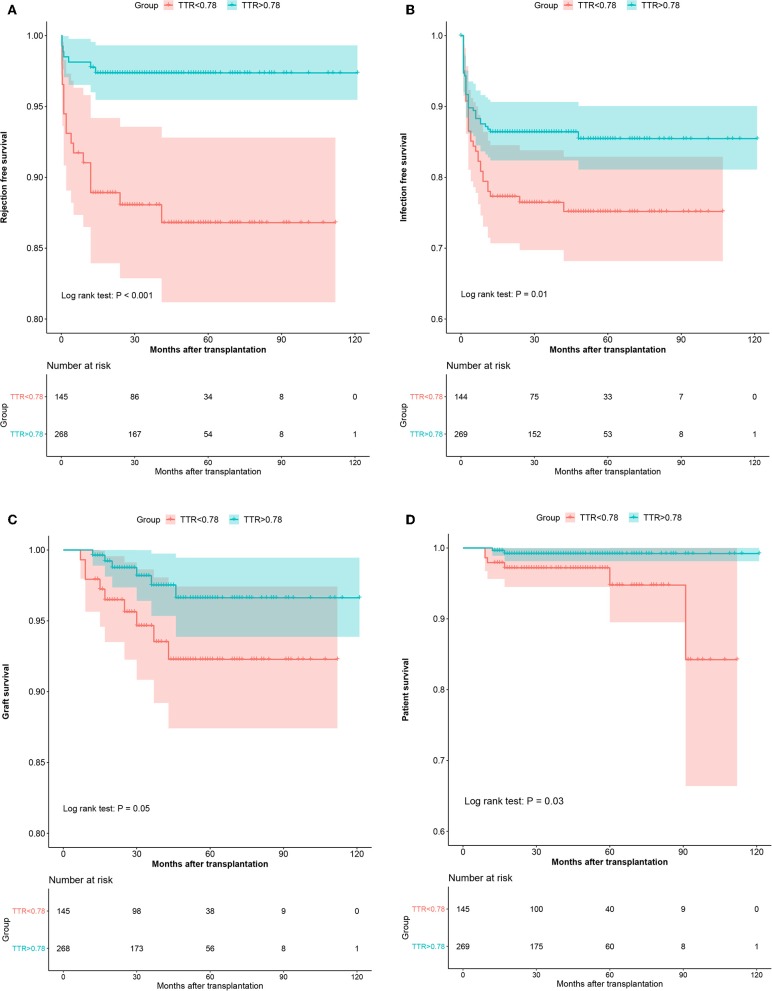
The Kaplan–Meier curves of the rejection-free survival **(A)**, infection-free survival **(B)**, graft survival **(C)**, and patient survival **(D)** in the validation cohort.

Similarly, in the validation cohort, more patients have experienced graft loss (6.2 vs. 2.6%, *P* = 0.069) in the low TTR group. Kaplan-Meier estimation indicated graft survival in high TTR group was significantly higher than that in low TTR group (Figure 4). Lower TTR was associated with a higher risk of graft loss (OR: 5.09, 95%CI: 1.28–23.65). Also, younger recipients (OR: 0.87, 95%CI: 0.78–0.96) and lower PRA (OR: 0.07; 95%CI: 0.01–0.56) were found to have independent protective effects on graft survival (Table 4).

**Table 4:** Univariate and Multivariate analysis of clinical characteristics for acute rejection, infection, and patient and graft survival in validation cohort.

**Characteristic**	**Acute rejection**				**Infection**				**Graft survival**				**Patient survival**			
	**Unadjusted**	***P***	**Adjusted**	***P***	**Unadjusted**	***P***	**Adjusted**	***P***	**Unadjusted**	***P***	**Adjusted**	***P***	**Unadjusted**	***P***	**Adjusted**	***P***
TTR	5.3 (2.16–13.03)	<0.001	5.36 (2.17–13.21)	<0.001	2 (1.19–3.34)	0.009	2 (1.19–3.34)	0.009	2.66 (0.95–7.46)	0.064	5.09 (1.28–23.65)	0.022	5.76 (1.15–28.93)	0.033	6.8 (1.34–34.61)	0.021
Donor age	1.01 (0.95–1.08)	0.731			0.99 (0.95–1.04)	0.751			0.95 (0.89–1.01)	0.085	0.93 (0.86–1)	0.066	0.92 (0.84–1.01)	0.086	0.91 (0.82–1.01)	0.089
Donor sex	0.64 (0.17–2.47)	0.521			0.81 (0.33–1.97)	0.643			0.69 (0.21–2.26)	0.538			/	/		
Recipient age	1 (0.95–1.05)	0.965			1 (0.97–1.03)	0.9			0.88 (0.8–0.96)	0.003	0.87 (0.78–0.96)	0.006	0.93 (0.83–1.03)	0.145		
Recipient sex	0.68 (0.29–1.59)	0.378			1.34 (0.74–2.42)	0.336			2.84 (0.64–12.71)	0.171			/	/		
Recipient BMI	0.91 (0.79–1.06)	0.222			1.01 (0.93–1.1)	0.769			1.02 (0.87–1.19)	0.815			1.01 (0.81–1.25)	0.957		
Time of dialysis	0.97 (0.93–1.01)	0.157			0.99 (0.98–1.01)	0.524			0.97 (0.91–1.03)	0.26			0.98 (0.92–1.05)	0.566		
DGF	/	/			/	/			/	/			/	/		
HLA mismatch	0.67 (0.22–2.04)	0.477			0.93 (0.43–2.02)	0.861			0.96 (0.21–4.35)	0.956			0.96 (0.12)	0.969		
PRA	0.27 (0.06–1.33)	0.109	0.25 (0.05–1.36)	0.109	2.14 (0.27–16.97)	0.472			0.16 (0.03–0.82)	0.028	0.07 (0.01–0.56)	0.013	0.18 (0.02–1.58)	0.12		
Cold ischemic time	1.14 (0.72–1.79)	0.575			1.06 (0.8–1.4)	0.704			0.92 (0.53–1.6)	0.759			0.97 (0.45–2.1)	0.936		
Induction therapy		0.334				0.578				0.347				0.237		
No																
ATG	1.85 (0.76–4.47)				0.9 (0.51–1.59)				2.23 (0.76–6.57)				4.16 (0.8–21.74)			
IL-2R	1.91 (0.57–6.37)				1.38 (0.65–2.94)				1.55 (0.3–7.94)				2.32 (0.21–26.14)			
Transplant year		0.21				0.73				0.32				0.63		
2007–2012	Reference				Reference				Reference				Reference			
2013–2017	0.76 (0.51–1.13)				0.96 (0.82–1.13)				0.85 (0.63–1.15)				0.92 (0.67–1.26)			

*ATG, antithymocyte globulin; BMI, body mass index; eGFR, estimated glomerular filtration rate; DGF, delayed graft function; HLA, human leukocyte antigen; PRA, panel reactive antibody; TTR, time in therapeutic range*.

### Recipient Survival

In the development cohort, during the follow-up, 7 (2.5%) and 2 deaths (0.4%) were recorded in the low TTR group and high TTR group (*P* = 0.016), respectively. The Kaplan–Meier estimation found that the high TTR group had significantly better patient survival than that of low TTR group (Figure 3D). The 3- and 5-year graft survival was 99.2 and 99.2% as well as 97.2 and 94.8% for the high and low TTR group, respectively. Lower TTR was an independent risk factor for graft loss in the multivariate analysis (OR: 6.54, 95%CI: 1.34–31.77) (Table 3).

In the validation cohort, a similar trend was observed with more patients experiencing patient death (4.1 vs. 0.7%, *P* = 0.043) in the low TTR group. Lower TTR was associated with a higher risk of patient death (OR: 6.8, 95%CI: 1.34–34.61) (Table 4). The Kaplan–Meier estimation indicated patient survival in the high TTR group was significantly higher (Figure 4).

### Acute Rejection

In the development cohort, 44 patients (15.5%) developed AR in the low TTR group compared to 32 (5.9%) in the high TTR group (*P* < 0.001). Patients without developing DGF (OR: 0.21, 95%CI: 0.06–0.77) were also observed to develop less AR. A multivariate analysis indicated that TTR ≤ 78% was an independent risk factor for AR (OR: 2.97, 95%CI: 1.82–4.84) (Table 3). Rejection-free survival was significantly higher in the high TTR group than that in the low TTR group (Figure 3).

In the validation group, similar results were observed. There were significantly more patients experiencing AR (12.4 vs. 2.6%, *P* < 0.001) in the low TTR group. Multivariate regression showed that TTR ≤ 78% was associated with a higher incidence of AR (OR: 2.97, 95%CI: 1.82–4.84) (Table 4). The Kaplan–Meier estimation indicated that rejection-free survival was significantly higher in the high TTR group than that in the low TTR group (Figure 4).

### Infection

For the development cohort, a total of 148 patients developed infection at least once with 63 (22.3%) in the low TTR group and 85 (15.6%) in the high TTR group (*P* = 0.018). Low TTR was associated with a higher risk of infection in the multivariate analysis (OR: 1.55, 95%CI: 1.08–2.22) (Table 3). The Kaplan–Meier estimation revealed that the infection-free survival was significantly higher in the high TTR group than that in the low TTR group (Figure 3).

Furthermore, in the validation group, we confirmed the results that more patients experienced infection episodes (24.1 vs. 13.8%, *P* = 0.008) in the low TTR group. TTR ≤ 78% was associated with a higher incidence of infection (OR: 2.00, 95%CI: 1.19–3.34) in the multivariate regression (Table 4). Infection-free survival in the Kaplan–Meier curve was significantly higher in the high TTR group than that in the low TTR group (Figure 4).

### Comparison to Other Tacrolimus Measures

Multivariate regression was also used to examine alternative measures for the characterization of tacrolimus exposure. We separated 1,241 patients into high and low TTR groups by TTR cut-off value. In 1,241 patients overall, increasing the TTR by 10% was associated with reduced patient death (OR = 0.73, 95%CI: 0.63–0.86) and graft loss (OR = 0.53, 95%CI: 0.36–0.80) (Table 5). Increasing IPV was an independent risk factor for graft loss, and all alternative tacrolimus measures were predictors for patient death. However, none of the tacrolimus measures were significantly associated with patient and graft survival in the high TTR group. In the low TTR group, increasing TTR by 10% remained independently protective for both patient (OR = 0.60, 95%CI: 0.40–0.93) and graft survival (OR = 0.51, 95%CI: 0.28–0.92). Only increasing IPV was associated with a higher incidence of graft loss (OR = 1.04, 95%CI: 1.01–1.07).

**Table 5:** Adjusted Cox regression models for patient and graft survival using different measures of tacrolimus exposure.

	**Overall patient**		**High TTR group**		**Low TTR group**	
	**Adjusted**	***P***	**Adjusted**	***P***	**Adjusted**	***P***
**GRAFT SURVIVAL**						
increasing TTR by 10%	0.73 (0.63–0.86)	<0.001	0.91 (0.44–1.91)	0.809	0.60 (0.40–0.93)	0.02
Increasing mean level	0.79 (0.55–1.13)	0.194	0.59 (0.26–1.36)	0.215	0.82 (0.45–1.50)	0.518
Increasing standard deviation	1.33 (0.66–2.54)	0.461	1.46 (0.06–37.04)	0.820	0.54 (0.14–2.09)	0.37
Increasing IPV%	1.05 (1.03–1.07)	<0.001	0.77 (0.48–1.24)	0.278	1.04 (1.01–1.07)	0.003
**PATIENT SURVIVAL**						
increasing TTR by 10%	0.53 (0.36–0.80)	0.002	1.16 (0.28–4.88)	0.836	0.51 (0.28–0.92)	0.026
Increasing mean level	2.49 (1.08–5.75)	0.032	1.79 (0.36–8.96)	0.476	2.44 (1.14–5.23)	0.021
Increasing standard deviation	2.49 (1.06–5.87)	0.037	0.94 (0.13–6.90)	0.952	2.70 (0.81–9.02)	0.107
Increasing IPV%	1.06 (1.00–1.13)	0.05	0.32 (0.003–28.87)	0.671	1.05 (0.96–1.16)	0.299

*IPV, intra-patient variability; TTR, time in therapeutic range*.

## Discussion

To the best of our knowledge, this is the first study to assess the effect of tacrolimus TTR on long-term outcomes in living kidney transplantation. In the current study, we found that a TTR above 78% was not only associated with improved graft survival but also with reduced risk of AR and infection. Tacrolimus TTR was more strongly associated with patient and graft survival than mean level, standard deviation (SD), and IPV.

The first reported use of TTR was for the therapeutic use of warfarin, a drug with considerable inter- and intra-patient variability and a narrow therapeutic index, much like tacrolimus. The warfarin TTR cut-off value was arbitrarily determined as 75% (10) or was recommended by European guidelines as being 70% or greater (18). Two studies investigating the use of tacrolimus TTR also arbitrarily set the TTR threshold as 30% in lung transplant (11) or 60% in kidney transplant (12). Although they found that increasing tacrolimus TTR was associated with improved clinical outcomes, the optimal TTR was not determined. The primary aim of maintaining the tacrolimus within the therapeutic range is to control rejection, and so we utilized the ROC curve to estimate the optimal TTR value based on the AR episodes in the first year. The cut-off value was 78%, and the AUC was 0.733. Though the AUC value was fair, the TTR of 78% can differentiate patients with increased risk of graft failure and patient death in the development group very well. Additionally, we further validated the TTR cut-off value and its role as an informative predictor in the validation cohort. Of note was the fact that, to accurately estimate TTR, we excluded cases with three or more consecutive missing values of tacrolimus. As the TTR was calculated by the Rosendaal method, which assumed a linear relationship exists between each measured value, that linear interpolation method was used to estimate the missing values (15). When there are three or more consecutive missing values, it is not reliable to apply interpolation, and this is due to the limitation of this technique.

We found that a higher tacrolimus TTR was strongly associated with better graft survival and rejection-free survival. These observations were consistent with the findings from previous kidney transplant and lung transplant analyses, which used the TTR to characterized tacrolimus exposure (11, 12). Notably, we did not find any difference in mean tacrolimus trough level between the high and low TTR groups. However, the high TTR group had much lower SD (1.9 vs. 1.4) and IPV (24.1 vs. 34.2%) of tacrolimus levels than that in low TTR group. Together with the facts that increasing SD and IPV of tacrolimus levels are independent risk factors for rejection and graft loss (6, 19), a higher TTR can thus predict better outcomes.

Interestingly, we found that TTR > 78% was associated with reduced infection, which was corroborated by the lung transplant study that found that increasing the TTR by 10% was associated with a decreased likelihood of infection (OR 0.81, 95%CI: 0.67–0.97) (11). We previously found that the increasing tacrolimus trough level at the first month was associated with infection, and those that had a tacrolimus trough level >7.15 ng/ml experienced a much higher incidence of infection (20). In the high TTR group, of 9,756 tacrolimus measures in 12 months, 8.0% had level >8 ng/ml, significantly lower than that in the low TTR group (14.4%, *p* < 0.001). Similar findings were confirmed in the first 3 months (3.2 vs. 6.4%, *p* < 0.001), indicating that the low TTR group had more patients with over-immunosuppression. Thus, the low TTR group may have a higher incidence of infection. However, when infection is diagnosed, a dose reduction is usually required. That erratic change of the tacrolimus level will result in a lower TTR.

The effects of several tacrolimus exposure characteristics have been investigated in kidney transplantation. A single measurement of an increased tacrolimus trough level soon after a kidney transplant has been associated with a decreased risk of AR and biopsy-proven AR (21, 22). However, a single time-point measurement may not be meaningful because tacrolimus trough levels are produced by dynamic rather than stable metabolic processes. Recently, a pooled analysis across four randomized trials found that the average tacrolimus level in first 12 months <4.0 ng/ml was associated with an increased incidence of BPAR (HR = 6.33, *p* < 0.00001) (7). Sapir-Pichhadze et al. conducted a retrospective cohort investigation of kidney transplants, examining the effect of the SD of tacrolimus levels on the composite endpoint (late allograft rejection, glomerulopathy, and total graft loss). They found that, for every 1-unit increase in SD, there was a 27% increase in the adjusted hazard of the composite endpoint (HR 1.27, 95%CI: 1.03–1.56) (6). Additional study using the mean level and SD to characterize tacrolimus exposure yielded similar findings (23, 24). Importantly, we found that the tacrolimus TTR was more strongly associated with patient and graft survival than mean level and SD. A subgroup analysis of the high TTR group found the predictive effect of all alternative measures disappeared. SD may poorly characterize levels that are consistently above or below the target range, and mean value cannot differentiate erratic changes between high and low levels, but TTR accounts for the concentration, variability, and the time between levels simultaneously. Together with our finding that a higher TTR can eliminate a large part of SD (1.4 vs. 1.9, *p* < 0.001), we found that that the TTR may better characterize exposure compared to the mean value and SD.

Increasing the IPV of tacrolimus is also a risk factor for inferior graft survival. In a retrospective analysis of 310 adult kidney transplants, an IPV >30% independently related to death censored graft loss (HR = 2.613, 95%CI: 1.361–5.016) and dnDSAs (HR = 2.925, 95%CI: 1.473–5.807) (25). However, we found the high TTR group had a much lower IPV than that in low TTR group (24 vs. 34%, *p* < 0.001), and tacrolimus TTR is more strongly associated with patient and graft survival than IPV, indicating that the TTR may be a better index of tacrolimus monitoring. What's more, the real-time clinical utility of IPV is limited because it cannot be generally computed at the bedside nor making clinical decisions based on the result. The ease of estimating TAC TTR supports its use as a risk-stratification and decision-making tool. Additionally, high IPV is mainly due to non-adherence (26), and the utilization of TTR may also stratify those with a high non-attendance rate in the outpatient clinic. As suggested by studies involving Warfarin, a less frequent dose change and better adherence contributed to a higher TTR level (27). Interventions to minimize the non-adherence, including the timing and dosing, may also improve tacrolimus TTR in kidney transplants and consequently better long-term allograft outcomes. In addition, a high-fat meal and administration of CYP3A4-interfering medications had a significant impact on the rate of TAC absorption and metabolism (28, 29), and so avoiding these factors may improve the TTR as well.

There are several limitations when interpreting our results. First, due to its retrospective nature, we could only establish an association between the TTR and the clinical outcomes. These findings should be confirmed with a prospective assessment. Second, tacrolimus was measured at discrete time points, and missing values were estimated by linear interpolation method. Though we excluded those with three or more consecutive missing values, estimated tacrolimus values may not have accurately reflected the real exposures. Third, the cut-off value of the TTR and its predictive role were based on our target range, whether the TTR remains associated with patient and graft survival in other tacrolimus target ranges was unknown. Finally, we only analyzed living kidney transplants; these observations therefore need to be externally validated in other transplant categories, such as deceased donor and ABO-incompatible kidney transplants.

## Conclusion

Tacrolimus TTR monitoring was predictive in achieving well-managed tacrolimus-based immunosuppression in living kidney transplants. Future prospective investigations should be conducted to confirm these findings.

## Data Availability Statement

The datasets analyzed in this article are not publicly available. Requests to access the datasets should be directed to kidney5@163.com.

## Ethics Statement

The studies involving human participants were reviewed and approved by the Ethical Committee of West China Hospital, Sichuan University. The patients/participants provided their written informed consent to participate in this study.

## Author Contributions

TS, SY, and TL participated in research design. TS and SY participated in the writing of the paper. YJ, ZH, JL, ZW, XL, and JZ participated in the data collection and data wash. YF, SY, TS, XW, and LL participated in data analysis.

### Conflict of Interest

The authors declare that the research was conducted in the absence of any commercial or financial relationships that could be construed as a potential conflict of interest.

## Supplementary Material

Supplementary Figure 1 A flow chart to describe the enrollment of patients.
