# Supplementary figures and images for: Increasing Time in Therapeutic Range of Tacrolimus in the First Year Predicts Better Outcomes in Living-Donor Kidney Transplantation
Source: Front Immunol. 2019 Dec 20;10:2912. doi: 10.3389/fimmu.2019.02912 (PMC6933438; doi:10.3389/fimmu.2019.02912)

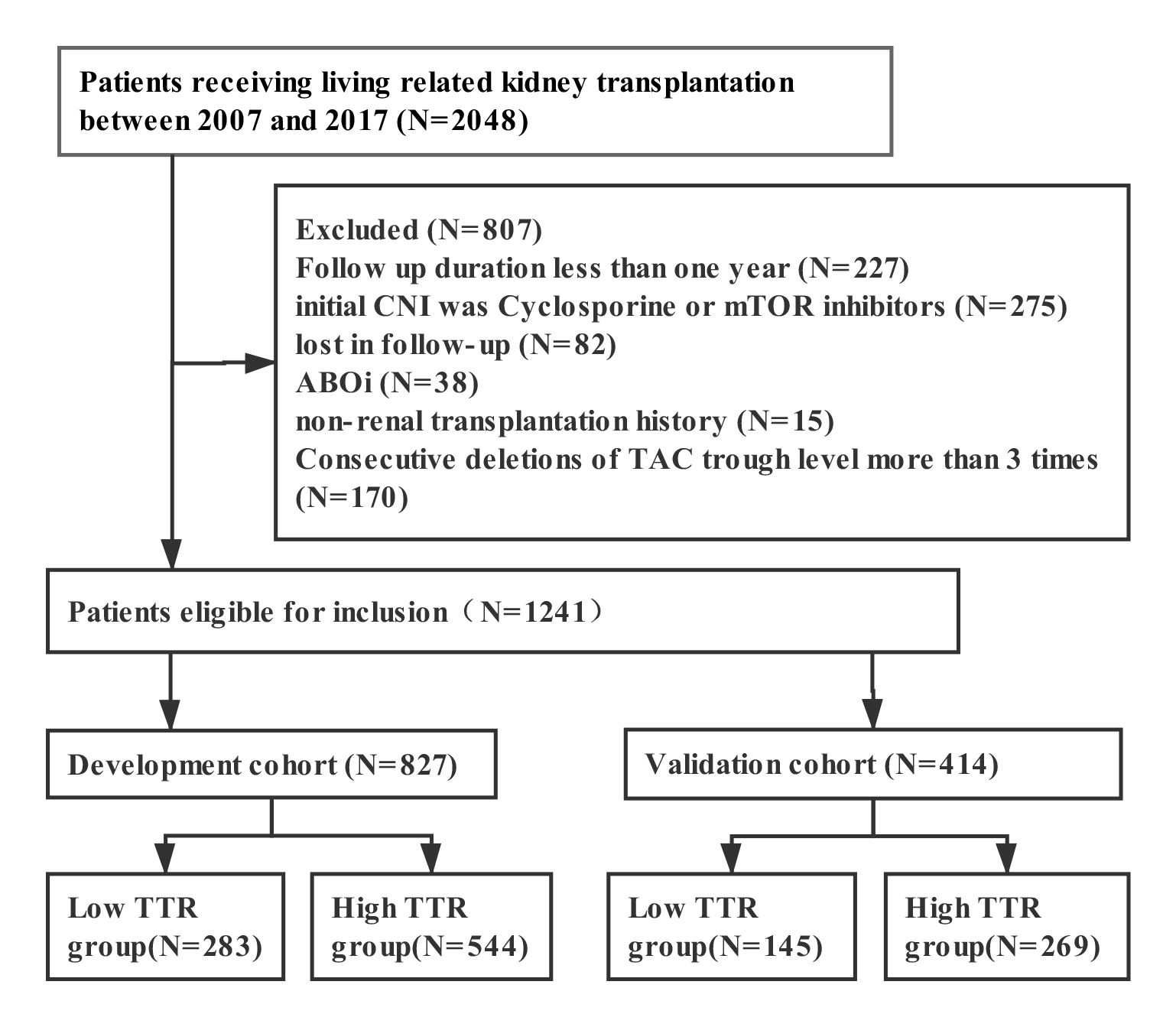

Supplement: Supplementary Figure 1 — A flow chart to describe the enrollment of patients. [file Image_1.TIF]
